# Genetic Characterization of *Rhizobium* spp. Strains in an Organic Field Pea (*Pisum sativum* L.) Field in Lithuania

**DOI:** 10.3390/plants13141888

**Published:** 2024-07-09

**Authors:** Justina Kaziūnienė, Francesco Pini, Arman Shamshitov, Kristyna Razbadauskienė, Birutė Frercks, Audrius Gegeckas, Raimonda Mažylytė, Laura Lapinskienė, Skaidrė Supronienė

**Affiliations:** 1 Institute of Agriculture, Lithuanian Research Centre for Agriculture and Forestry, LT-58344 Akademija, Lithuaniaskaidre.suproniene@lammc.lt (S.S.); 2 Department of Biology, University of Bari Aldo Moro, 70125 Bari, Italy; 3 Institute of Horticulture, Lithuanian Research Centre for Agriculture and Forestry, Kaunas Str. 30, LT-54333 Babtai, Lithuania; 4 Life Sciences Center, Institute of Biosciences, Vilnius University, LT-10257 Vilnius, Lithuania; 5 Department of Biology, Faculty of Natural Sciences, Vytautas Magnus University, 53361 Kaunas, Lithuania

**Keywords:** *Rhizobium* spp., *Pisum sativum* L., pea, 16 S rRNA, *recA*, *atpD*, *nodC*

## Abstract

Biological nitrogen fixation in legume plants depends on the diversity of rhizobia present in the soil. Rhizobial strains exhibit specificity towards host plants and vary in their capacity to fix nitrogen. The increasing interest in rhizobia diversity has prompted studies of their phylogenetic relations. Molecular identification of *Rhizobium* is quite complex, requiring multiple gene markers to be analysed to distinguish strains at the species level or to predict their host plant. In this research, 50 rhizobia isolates were obtained from the root nodules of five different *Pisum sativum* L. genotypes (“Bagoo”, “Respect”, “Astronaute”, “Lina DS”, and “Egle DS”). All genotypes were growing in the same field, where ecological farming practices were applied, and no commercial rhizobia inoculants were used. The influence of rhizobial isolates on pea root nodulation and dry biomass accumulation was determined. 16S rRNA gene, two housekeeping genes *recA* and *atpD,* and symbiotic gene *nodC* were analysed to characterize rhizobia population. The phylogenetic analysis of 16S rRNA gene sequences showed that 46 isolates were linked to *Rhizobium leguminosarum*; species complex 1 isolate was identified as *Rhizobium nepotum*, and the remaining 3 isolates belonged to *Rahnella* spp., *Paenarthrobacter* spp., and *Peribacillus* spp. genera. *RecA* and *atpD* gene analysis showed that the 46 isolates identified as *R. leguminosarum* clustered into three genospecies groups (B), (E) and (K). Isolates that had the highest influence on plant dry biomass accumulation clustered into the (B) group. *NodC* gene phylogenetic analysis clustered 46 *R. leguminosarum* isolates into 10 groups, and all isolates were assigned to the *R. leguminosarum* sv. *viciae*.

## 1. Introduction

Field pea (*P. sativum* L.) is one of the most popular legume plants in crop rotations in Lithuania [1,2]. Legume plants’ inclusion in rotations offers a multitude of advantages which are significant to agricultural productivity and sustainability. Legume plants’ cultivation enhances soil quality through various mechanisms. These include the improvement of soil structure, which promotes better water infiltration and retention [3,4]. It also breaks pest cycles and makes weed control easier [5,6,7]. Additionally, legume plants such as field peas improve the growth of soil microbial biomass which plays a crucial role in nutrient cycling and overall soil fertility [8]. One of the most significant benefits of incorporating legume crops into rotations is their ability to form symbiosis with rhizobia, fix atmospheric nitrogen and increase the nitrogen accumulation in the soil. This natural process reduces the need for mineral nitrogen fertilizers in subsequent non-legume crops [6,7,9]. In general, the integration of field pea or other legume plants into crop rotations helps implement the European Union (EU)’s strategies of “Bringing nature back into our lives” [10] and “The Farm to Fork” [11], which aim to reduce pesticides and fertilizers in European agriculture.

The amount of nitrogen fixed by pea plants can vary due to many factors such as soil’s chemical and physical properties, environmental conditions, weed and insect infestation, crop rotation and the rhizobia population [12,13,14,15]. Rhizobia population can vary due to the same factors listed above, however, biotic factors, such as the competitiveness of microorganisms and commercial inoculants, affect the *Rhizobium* population as well [16,17]. *Rhizobium* spp. includes more than 90 species, which can nodulate different legume plants [18]. The *R. leguminosarum* species was initially regarded as the sole species able to establish symbiosis with *P. sativum*. *R. leguminosarum* includes different symbiovars nodulating different legume plants. For example, *R. leguminosarum* sv. *viciae* forms a symbiosis with plants of the Viciaea tribe, like vetches (*Vicia* L.) and pea (*P. sativum* L.), *R. leguminosarum sv. trifolii* nodulates clovers (i.e., *Trifolium* L.), and *R. leguminosarum sv. phaesolii* infects common beans (*Phaseolus vulgaris* L.) [18]. However, more species were introduced as symbionts of the legume tribe *Vicieae*, such as *R. fabae* [19], *R. pisi* [20], *R. lentis* [21], *R. binae* [21], *R. bangladeshense* [21], and *R. anhuiense* [22].

The population of rhizobiain pea fields remains poorly documented, especially in Lithuania and under organic farming conditions. This study aimed to analyze the rhizobia population and genetically characterize rhizobial strains isolated from different organically grown pea genotypes, and evaluate the strain impact on plant nodulation and biomass accumulation. Five different pea genotypes selected for a wide range rhizobia isolation: “Respect”, “Astronaute”, “Bagoo”, “Lina DS” and “Egle DS” were characterized by abundant grain yield, resistance to loading and disease infection. Lina DS” and “Egle DS” are new and less studied Lithuanian pea genotypes listed in national and EU variety lists in 2021. Different genes 16S rRNA, atpD, recA and nodC were analysed for rhizobial isolates’ genetic identification.

## 2. Results

### 2.1. Rhizobial Strain Isolation, Screening and Evaluation of Nodulation and Plant Biomass Accumulation

**Figure 1:**
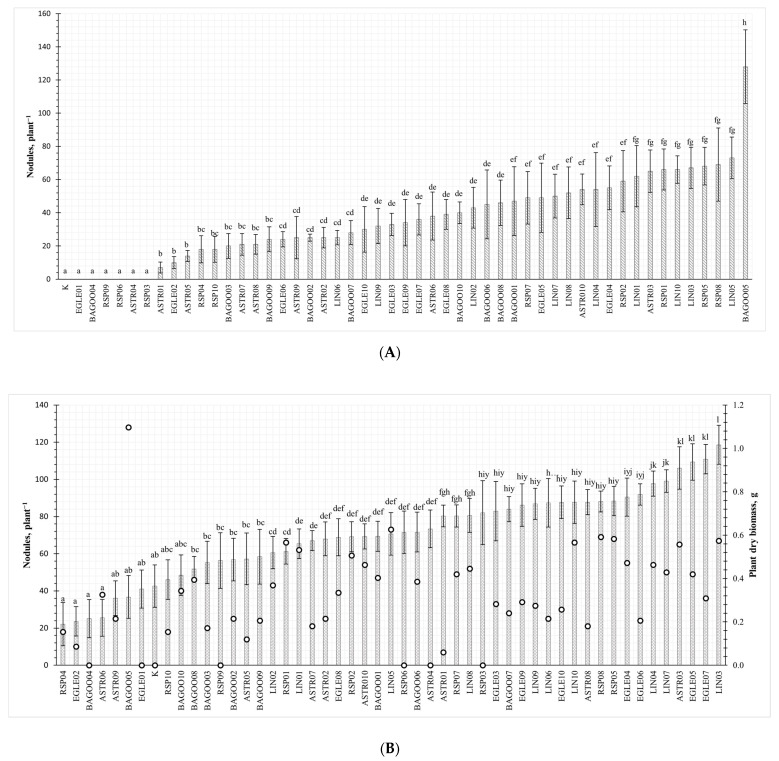
(**A**). The average number of nodules on *P. sativum* roots after inoculation with different rhizobia isolates. Error bars indicate the standard deviations within biological replications at each treatment. Values marked with the same letter are not significantly different at *p* ≥ 0.05. (**B**). The averages of the plant dry biomass (columns) and number of nodules on *P. sativum* roots (ovals) after inoculation with different rhizobia isolate. Error bars indicate the standard deviations within biological replications at each treatment. Values marked with the same letter are not significantly different at *p* ≥ 0.05.

Fifty isolates obtained from root nodules of five pea genotypes exhibited morphological and biochemical features typical to rhizobial strains. All isolates were Gram negative rods of circular form, convex elevation, entire margin and which formed cream–white colonies. A nodulation test with rhizobia isolates and pea genotype “Egle DS” showed that RSP03, RSP06, RSP09, ASTR04, EGLE01 and BAGOO04 isolates did not form nodules on peas roots (Figure 1A,B). RSP03, RSP06 and RSP09 were isolated from pea genotype “Respect”, and isolates ASTR04, EGLE01 and BAGOO04 were isolated from “Astronaute”, “Egle DS” and “Bagoo” genotypes, respectively. In control plants, no nodules were found. The significantly highest number of nodules (128 units per plant) was formed on the plants where isolate BAGOO05 was applied. In other variants, the average number of nodules varied from 7 to 73 units per plant.

Inoculation of rhizobia isolates had different effects on plant biomass formation. Only 35 rhizobia isolates significantly increased dry pea biomass compared to the non-inoculated control. The highest plant biomass was achieved when LIN03, EGLE07, EGLE05 and ASTR03 isolates were inoculated, and the results were 2.8-fold, 2.6-fold, 2.6-fold and 2.5-fold higher compared to the control. No significant differences between the control and plants inoculated with RSP04, EGLE02, BAGOO04, ASTR09, BAGOO5, EGLE01, RSP10, BAGOO10, BAGOO08, BAGOO03, RSP09, BAGOO2, ASTR05 and BAGOO09 strains were detected.

**Figure 2:**
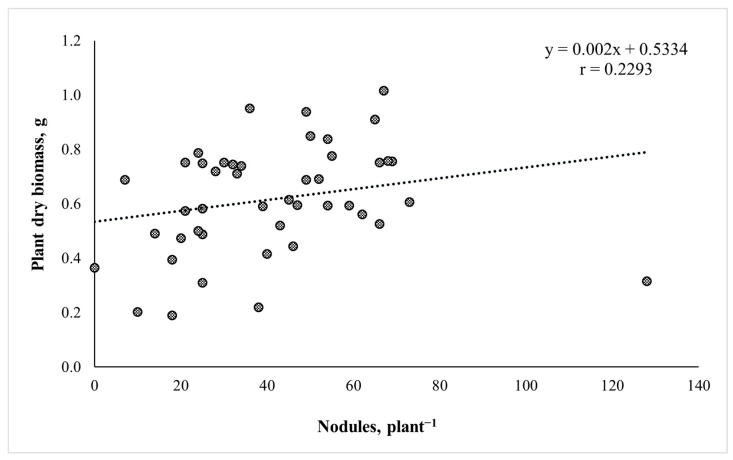
Correlation between number of nodules and dry plant biomass, *p* < 0.05.

A correlation analysis was carried out to investigate the relationship between number of nodules and dry plant biomass (Figure 2). The results indicated a positive and significant correlation (*p* < 0.05) between number of nodules and dry plant biomass accumulation. However, the strength of the correlation was weak, with a correlation coefficient of r = 0.2293.

### 2.2. Phylogenetic Analysis of 16s rRNA, recA, atpD and NodC Gene Sequences

**Figure 3:**
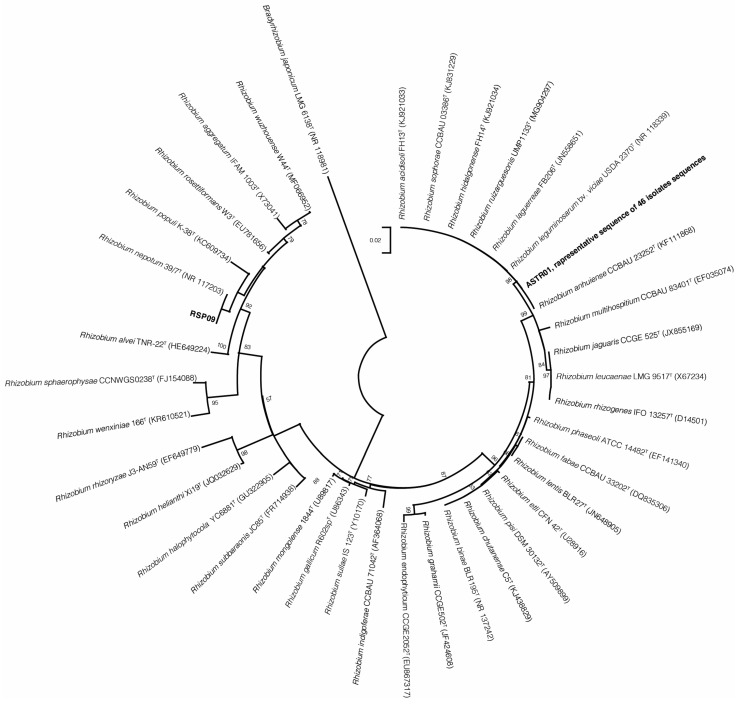
The phylogenetic tree of 16S rRNA gene sequences of the *P. sativum* root nodule isolates (shown in bold), related strain types of *Rhizobium* bacteria and reference *Bradyrhizobium japonicum* LMG 6138; accession numbers indicated in brackets. *B. japonicum* LMG 6138 was used as an outgroup to root the tree. The evolutionary history was inferred by using the Maximum Likelihood algorithm with the Kimura 2-parameter model. There were a total of 642 positions in the final dataset.

A phylogenetic tree based on 16S rRNA gene sequences was constructed to analyze the diversity of bacterial strains isolated from organically grown peas (Figure 3). In total, 46 isolates, including nodules not forming isolates, such as EGLE01, BAGOO04, were attributed to the *R. leguminosarum* group. Isolate RSP09 was identical to the *R. nepotum* 39/7, and the remaining three isolates did not belong to *Rhizobium* spp. Isolates ASTR04, RSP03 and RSP06 were grouped into *Rahnella* spp., *Peribacillus* spp. and *Paenarthrobacter* spp. genera, respectively, and were not shown in the phylogenetic tree of 16S rRNA gene sequences.

**Figure 4:**
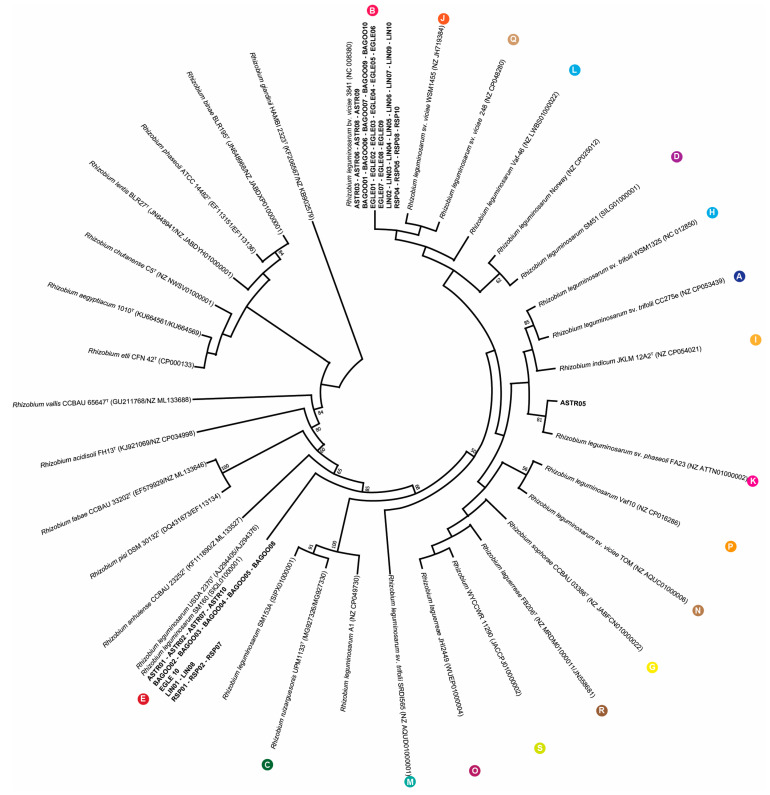
The phylogenetic tree based on the concatenated alignment of *recA* and *atpD* gene sequences of the *Rhizobium* spp. root nodule isolates (shown in bold) and related strain of *Rhizobium* bacteria. The evolutionary history was inferred by using the Maximum Likelihood algorithm with the Tamura-Nei model. There were a total of 904 positions in the final dataset. Letters in colored circles indicate the different *R. leguminosarum* genospecies.

The *recA* and *atpD* gene sequences of all 47 *Rhizobium* spp. isolates were analysed to attribute each isolate to one of the 18 different genospecies. The phylogenetic analysis of *recA* and *atpD* gene sequences confirmed 16s rRNA gene sequencing results that RSP09 isolate belongs to *R. nepotum*. It also revealed that all 46 isolates, even non-nodulating EGLE01 and BAGOO04 isolates can be identified within the *R. leguminosarum* complex. Analysis of *atpD* and *recA* gene sequences allowed the different *Rhizobium* spp. isolates by genospecies to cluster (Figure 4). *Rhizobium* spp. isolates divided into three groups: 30 isolates clustered with *R. leguminosarum* bv. *viciae* 3841 (genospecies B), 15 isolates formed close clustering with *R. leguminosarum* USDA 2370T (genospecies E) and only the ASTR05 isolate was clustered to *R. leguminosarum* sv. *phaseoli* FA23 (genospecies K). The most similar isolates fell into the same cluster. Almost all isolates isolated from “Egle DS” and “Lina DS” genotypes clustered within genospecies B, the only one EGLE10 isolate and two LIN01 and LIN08 isolates were assigned within the genospecies E. *R. leguminosarum* isolates from “Bagoo”, “Astronaute” and “Respect” genotypes dispersed into K as well as B genospecies.

**Figure 5:**
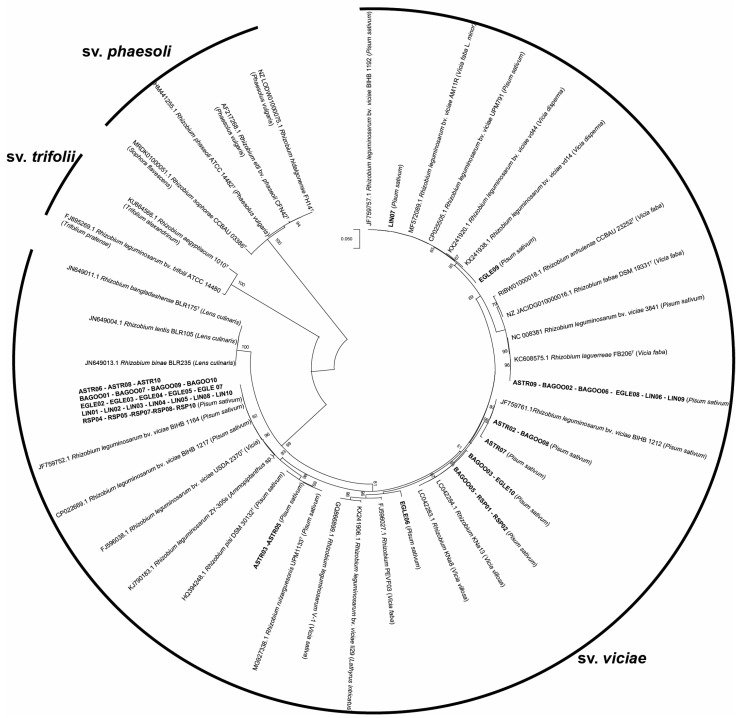
The phylogenetic tree of *nodC* gene sequences of the *R. leguminosarum* root nodule isolates. The phylogenetic tree is based on the alignment of the *nodC* gene sequences (from position 337 to 1015 for a total of 679 nucleotides). The tree is inferred using the maximum likelihood algorithm with the Tamura 3 parameter model. Bootstrap values (>50%) are shown next to the branches. Sequences of the rhizobia strains used in this work are highlighted in bold for those strains showing identical *nodC* sequences; representative sequences for each group have been used.

Not all *R. leguminosarum* isolates’ *nodC* gene amplification was successful. *NodC* gene amplification of EGLE01, BAGOO04 and ASTRO01 isolates failed, so these isolates were not included in the *NodC* gene phylogenetic tree. Based on the phylogenetic analysis of the *NodC* gene, 43 *R. leguminosarum* isolates clustered into 10 different groups (Figure 5). The most genetically related strains were clustered in the same group. *R. leguminosarum* bv. *viciae* BIHB 1164 group was the most filled, and it included 24 *R. leguminosarum* strains isolated from all five pea genotypes. This group included 20 *R. leguminosarum* isolates from genospecies B and only 4 isolates from genospecies E. Other *R. leguminosarum* strains spread into smaller groups. *R. leguminosarum* bv. *viciae* BIHB 1192 and *R. leguminosarum* bv. *viciae* vd14 clustered with LIN07 and EGLE09 isolates, which both belonged to the same genospecies B. Also, five isolates assigned to the *R. larguerreae* FB206 cluster belonged to the genospecies B group, and only one isolate, BAGOO02, belonged to the genospecies E group. Instead, all isolates in *R. leguminosarum* bv. *viciae* BIHB1212 and *R.* KNa13 clusters were from genospecies E. Lastly, one isolate from B and one isolate from K genospecies clustered with *R. ruizaguesonis* UPM1133. However, all *R. leguminosarum* isolates were attributed to *R. leguminosarum* sv. *viciae* symbiovar.

## 3. Discussion

The 50 bacterial isolates were isolated from five different pea genotypes, which were growing in the same field. Soil chemical analysis showed that the humus content in soil was low at 2.84%, however, other soil parameters were suitable for pea growth and nutrient assimilation [23,24]. Mobile phosphorus and potassium quantity in analysed soil was high, and soil pH was near neutral, so these minerals were not fixated into insoluble compounds and they were available for the peas [25]. Mineral nitrogen content in the soil was not high at 9.82 mg kg^−1^ or 47.1 kg ha^−1^ [26]. This amount of nitrogen should have less or no negative impact on the nodulation of different pea genotypes.

A pea nodulation test showed that not all isolates formed nodules repeatedly. No nodules were formed on pea roots where isolates RSP03, RSP06, RSP09, ASTR04, EGLE01 and BAGOO04 were inoculated, however, plant biomass analysis showed that isolates ASTR04, RSP03 and RSP06 had a significant positive influence on dry plant biomass formation. Genetic analyses revealed that these three isolates ASTR04, RSP03 and RSP06, isolated from “Astronaute” and “Respect” genotypes, belonged to *Rahnella* spp., *Peribacillus* spp. and *Paenarthrobacter* spp. genera, respectively. However, some research has demonstrated that these three genera members could be beneficial for legume plants on the different plant growth-stimulating mechanisms [27,28,29]. For example, *Rahnella* sp. BIHB 783 isolate is described as a cold-adapted psychrotroph which produces indole-3-acetic acid, indole-3-acetaldehyde, indole-3-acetamide, indole-3-acetonitrile, indole-3-lactic acid and other plant beneficent compounds. This strain also has the ability to solubilize organic and inorganic phosphorus forms. In the same study, *Rahnella* sp. BIHB 783 isolate inoculation significantly increased pea shoot length, dry weight and yield by 25%, 32% and 37%, respectively [27]. We found a few studies where scientists demonstrated that pea lateral root number increased significantly after *Peribacillus simplex* 30N-5 inoculation [28]. Also, the fresh weight of soybean root was significantly improved and was two times higher after *P. simplex* R180 inoculation [29]. *Paenarthrobacter nitroguajacolicus* UP1 isolated from pea rhizosphere showed phosphorus and potassium solubilization activity and siderophore production in in vitro experiments [29]. Our results showed that the tree isolates ASTR04, RSP03 and RSP06 increased dry plant biomass significantly compared to the control. The average dry plant biomass increased 1.7-fold where isolates ASTR04 and RSP06 were inoculated, and 1.9-fold where isolate RSP03 was inoculated. Plants inoculated with isolate RSP03 accumulated significantly higher biomass compared to ASTR04- and RSP06-inoculated plants. It is possible that these isolates’ consortium with rhizobia could improve legume plant growth and development, however, additional plant analysis is necessary to confirm that presumption.

Phylogenetic 16S rRNA gene sequence analysis showed that 47 isolates were assigned to *Rhizobium* spp., among them RSP09, EGLE01 and BAGOO04 isolates that did not form nodules. RSP09 isolate clustered with *R. nepotum* 39/7 type strain. *R. nepotum* belongs to a phylogenetic clade formerly known as agrobacteria and is considered as non-symbiotic and nonnodulating bacteria [30]. Few studies reported that *R. nepotum* can fix nitrogen, and produce auxins and siderophore [31,32]. Another work observed that *R. nepotum* inoculation had a positive effect on soybean leaf area, pod weight and number of pods [33], however, this bacterial species is less studied with plants. In our research, no significant differences in average dry plant biomass formation were observed between RSP09-inoculated plants and the control variant. Phylogenetic analysis of 16S rRNA gene sequences revealed that 46 isolates, including nodules not formed from isolates EGLE01 and BAGOO04, were attributed to. the *R. leguminosarum* group, which included *R. leguminosarum* bv. *viciae* USDA 2370, *R. laguerreae* FB206, *R. sophorae* CCBAU 03386, *R. anhuiense* CCBAU 23252, *R. acidisoli* FH13, *R. ruizarguesonis* UMP1133 and *R. hidalgonense* FH14 type strains. According to the scientific literature, 16S rRNA gene sequences of these type strains in the *R. leguminosarum* group are similar in 99.9% of cases [34,35], therefore, it is problematic to determine isolates’ phylogeny with a specific species in this cluster. Phylogenetic analysis of *Rhizobium* spp. 16S rRNA sequences is not indicative of genospecies [35].

According to scientific research, *R. leguminosarum* is too diverse to be considered a single species, and it can be described as a species complex [36]. Kumar et al. were the first to divide the *R. leguminosarum* isolates into five genospecies A–E [36]. The *R. leguminosarum* isolates were separated by average nucleotide identity (ANI), and values below 95%, represent separate species [37]. Recently extensive genomic analysis has subdivided *R. leguminosarum* into 18 different genospecies, 5 of which include the type strains of named species: *R. laguerreae*, *R. sophorae*, *R. ruizarguesonis*, *R. indicum* and *R. leguminosarum* itself [35]. The results of our performed phylogenetic *atpD* and *recA* gene analysis showed that 46 *Rhizobium* spp. isolates divided into three genospecies: 30 isolates clustered with *R. leguminosarum* bv. *viciae* 3841 (genospecies B), 15 isolates formed close clustering with *R. leguminosarum* USDA 2370T (genospecies E) and only one ASTR05 isolate was clustered to *R. leguminosarum* sv. *phaseoli* FA23 (genospecies K). All 46 isolates were identified as *R. leguminosrum*. Most of *Rhizobium* spp. isolates isolated from “Egle DS” and “Lina DS” genotypes clustered within the same genospieces (B). According to the Lithuanian Research Centre for Agriculture and Forestry (LAMMC), breeders “Egle DS” and “Lina DS” are related genotypes. “Egle DS” was developed by crossbreed “Respect” and “Audit” genotypes, and “Lina DS” was developed by crossbreed “Grafila” and “Respect”. However, *R. leguminosarum* isolates isolated from “Respect”, “Bagoo” and “Astronaute” genotypes dispersed into (B) and (E) clusters evenly. Several studies indicated that nodulation and BNF are influenced not only by the population of rhizobia in the soil but also by the genotype of the legume plant. Different legume genotypes can attract different rhizobia strains, while the same rhizobia strain can fix different amounts of nitrogen in different plant genotypes [38,39,40]. Those differences are associated with specific flavonoids produced by different legume genotypes responsible for rhizobia attraction to the root hairs. Rhizobia, in turn, produces the nod factors that induce morphological changes in their host plant root system [41]. This means that symbiosis formation is controlled by multiple genetic factors from pea and rhizobia [42]. It is possible that “Egle DS” and “Lina DS” genotypes produce similar flavonoids and attract genetically similar rhizobia strains. Another option is that genetically related rhizobia produce similar nod factors which are easily recognized by related genotypes.

Currently, the *nodC* gene is commonly used as a phylogenetic marker of *Rhizobium* spp. symbiovars [43]. The reason is that *nod* genes encode species-specific modifications to the nod factor structure and, related to this, specific *nod* genes have been shown to be major determinants of legume host specificity [44,45,46]. *NodC* gene analysis showed that 43 *R. leguminosarum* isolates spread into 10 different groups and were assigned to *R. leguminosarum* sv. *viciae* symbiovar. This also revealed that some isolates from different genospecies have the same *nodC* genes and are clustered in the same groups. Some studies suggest that *nod* and *nif* genes are symbiotic, adaptive genes and, in many cases, have an evolutionary history independent of the rest of the genome. *Nod* loci could be transferred by lateral gene transfer, even across divergent chromosomal lineages [47]. Horizontal transfer of nodulation genes can potentially help rhizobia adapt to a new host plant or enable genetically different bacteria with similar *nod* genes to form symbiotic interactions with the same legume plants [47,48]. Phylogenetic analysis results confirmed nodulation results, i.e., that these isolates form a symbiosis with pea plants. The same 43 isolates formed nodules on Lithuanian pea genotype “Egle DS” roots. However, *nodC* gene amplification of EGLE01, BAGOO04 and ASTRO01 isolates was not successful. Amplification of these isolates was repeated five times, different PCA conditions were applied, but the *nodC* gene was not amplified. *AtpD* and *recA* gene analysis of EGLE01, BAGOO04 and ASTRO01 revealed that these isolates can be identified as *R. leguminosarum*. Nodulation test results indicated that the ASTRO01 isolate can form nodules on pea roots, while EGLE01, and BAGOO04 do not form nodules and symbiosis with pea plants. Whereas *nodC* gene amplification with these isolates failed, we can only presume that ASTRO01 could have been assigned to *R. leguminosarum* sv. *viciae* symbiovar, and that the remaining two EGLE01 and BAGOO04 isolates may potentially be categorized into other symbiovars, which are incapable of forming a symbiosis with pea plants. However, *nodC* gene phylogenetic analysis results of 43 *R. leguminosarum* isolates confirmed that this gene could be used for symbiotic relationship determination between *R. leguminosarum* isolates and different legume plants [49,50].

Nitrogen-fixing rhizobia strains increase biomass accumulation in legumes, and consequently dry plant biomass serves as a pertinent indicator for the potential evaluation of rhizobia nitrogen fixation [51,52]. Pea biomass analysis with the “Egle DS” genotype revealed that 17 out of 18 rhizobia isolates, which were the most effective on dry plant biomass formation, belonged to the same genospieces (B), and only the isolate EGLE10 grouped in the cluster (E). Most of the dry plant biomass-increasing isolates were isolated from Lithuanian pea genotypes, and seven rhizobia isolates were obtained from “Egle DS”, while six strains were obtained from “Lina DS”. This revealed that the highest plant biomass was achieved when isolates of the same genotype or related “Lina DS” genotype were used for plant mono-inoculation. These results could be important for highly effective commercial *Rhizobium* inoculate formulation and adaptation for specific pea genotypes. The nodulation results showed the most nodulating rhizobia isolates dispersed similarly into both (B) and (E) clusters. Few of the most nodulated RSP08, RSP05, LIN03, LIN10, ASTR03, EGLE04, LIN04, LIN07 and EGLE05 isolates also exhibited high efficiency on plant biomass accumulation. However, some of rhizobia isolates formed a high nodule number, but had less or no effect on plant biomass formation. Correlation analysis revealed that the relationship between nodulation and dry plant biomass accumulation was significantly positive, but weak (r = 0.2293). The weak correlation suggests that, while dry plant biomass tends to increase with the number of nodules, the nodulation is not the sole determinant of plant biomass accumulation. The growth of a plant and its biomass accumulation are complex processes that depend on many external and internal factors. These findings confirmed the results of the other authors, indicating that a high nodule number is an important parameter demonstrating rhizobia nodulation ability, however, this does not guarantee biomass accumulation or efficient nitrogen fixation for the plant. Plant biomass formation and nitrogen accumulation depend on plant genetic characteristics, soil composition, climate and rhizobia–strain–nitrogen–fixation efficiency [53,54]. This has scientifically proven that legumes’ symbiosis with partially efficient or inefficient rhizobia strains can interfere with root nodulation through efficient rhizobia strains, consequently reducing biological nitrogen fixation for the plant [40,55,56].

In summary, phylogenetic analysis of *recA and atpD* genes showed that rhizobia diversity in the field where ecological farming was applied and no commercial rhizobia inoculants were used, was not high, and most of the isolates were genetically similar and clustered in two main clusters (B) and (E), and only one isolate was attributed to (K) cluster. Nevertheless, most of the isolates significantly increased pea biomass accumulation and intensively formed nodules. Many studies have shown that rhizobia inoculants can improve nodulation, nitrogen fixation and grain yield [57,58,59,60], however, highly effective and competitive rhizobia strains need to be selected to ensure efficient BNF [61,62]. Our isolated and analysed LIN03, EGLE05 and ASTR03 rhizobia strains have high potential as biofertilizers, and these isolates formed a high number of nodules and increased pea biomass more than 2.5-fold compared to the control. However, nitrogen fixation efficiency, competitiveness and plant reinoculation with the same and different genotype strains should additionally be analysed in further investigations to create highly effective *Rhizobium* inoculates for specific pea genotypes or wide-range applications.

## 4. Materials and Methods

### 4.1. Sample Collection

Soil samples and *P. sativum* L. (field pea) seedlings were collected in 2022 in the Kedainiai district, Dotnuva [55.396450, 23.866748]. Pea seedlings were collected at the stem elongation stage (BBCH 35) from one field where five different pea genotypes were grown in 10 × 1.5 m blocks next to each other. Three pea genotypes commonly grown in Lithuania (“Bagoo”, “Respect” and “Astronaute”) and two new genotypes bred by LAMMC (Lina DS and Egle DS) were chosen to isolate rhizobia. Ten plants from each pea genotype were collected for rhizobia isolation.

Soil samples were collected in the same field where field pea samples were collected. Commercial rhizobia inoculants had never been used in this field before. Fifteen soil samples were collected randomly at a depth of 0–30 cm [63]. Samples were sieved through a 5-mm pore sieve, combined into one sample and stored at +4 °C. Chemical (pH, N, P_2_O_5_ and K_2_O) and physical (granulometric composition and humus) analyses of the combined soil sample were performed (Table 1).

### 4.2. Isolation and Screening of Rhizobia

First, ten pink-pigmented nodules, each larger than 2 mm in diameter, were selected from the roots of ten plants of the same genotype, with one nodule chosen from each plant. All nodules from the same pea genotype were washed together with sterile deionized water (SDW) and sterilized in 95% (*v*/*v*) ethyl alcohol solution for 10 s, then sterilized in 3% sodium hypochlorite solution for 3 min and washed five times with SDW. Each sterile nodule was transferred to a different tube with 2 mL SDW water and homogenized using a different sterile glass rod for each nodule suspension. The prepared bacterial suspensions were diluted in 10^−3^, 10^−4^ and 10^−5^ series according to the serial dilution method, and 100 µL of each dilution was plated onto solid yeast extract mannitol (YEMA) [0.5 g/L K_2_HPO_4_, 0.2 g/L MgSO_4_, 0.1 g/L NaCl, 1.0 g/L CaCO_3_, mannitol 10.0 g/L, yeast extract 1.0 g/L, agar 20 g/L] media. Plates were incubated in a microbiological incubator at 28 °C for 48 h [64]. Strains were purified, streaking a single colony on new YEMA plates at least five times until only morphologically identical cells remained in the strained biomass. Purified rhizobia strain suspensions were frozen in 20% (*v*/*v*) glycerol solution at −80 °C.

All 50 isolates were characterized for selected morphological properties such as colony shape, border, size, elevation, color, transparency, mucosity [65] and capacity to produce the exopolysaccharide gum [66]. Gram staining was performed as described by [67].

### 4.3. Pea Analysis with Different Rhizobia Isolates

The nodulation ability of rhizobia isolates was tested through experiments with *P. sativum* plants. Three-year field experiments demonstrated that “Egle DS” was the most nodulating and the most productive genotype among all five examined pea genotypes. Based on those results, the “Egle DS” genotype was selected for the nodulation test. Pea seeds were sterilized in a 70% ethyl alcohol solution, washed with SDW and additionally disinfected with sodium hypochlorite solution containing 5% active chlorine for 5 min. Finally, sterile seeds were rinsed 5 times with SDW. Sterile seeds were germinated at room temperature, in the dark, for 4 days. One pea seedling was planted in a 500 mL vegetative pot which was filled with a sterile mixture of vermiculite and sand in a ratio of 3:1 and supplied with 100 mL of a nitrogen-free nutrient medium [1 mM CaCl_2_ × 2H_2_O, 0.1 mM KCl, 0.8 mM MgSO_4_ × 7H_2_O, 10 μM Fe EDTA, 35 μM H_3_BO_3_, 9 μM MnCl_2_ × 4H_2_O, 0.8 μM ZnCl_2_, 0.5 μM Na_2_MoO × 2H_2_O, 0.3 μM CuSO_4_ × 5H_2_O, 3.68 mM KH_2_PO_4_ and 4 mM Na_2_HPO_4_, pH = 6.5] [68].

Rhizobia isolates were grown in TY broth [5 g tryptone, 3 g yeast extract and 1.3 g CaCl_2_ × 6H_2_O] [69] for 24 h until optical density (OD600) reached 0.6–0.8. Then, isolate suspensions OD600 were adjusted to =0.05 using sterile 0.8% NaCl solution. The number of cells in 1 mL of prepared rhizobia isolate suspension was obtained according to the serial dilution method, plating suspensions on a solid YEMA medium [2]. Pea seedlings at the leaf development stage (BBCH 07) were inoculated with 1 mL rhizobia isolate suspension and grown in the plant growth chamber for 1 month (BBCH 37) using a 16/8-h day/night photoperiod and 20/16 °C day/night temperature modes [70]. Non-inoculated plants were used as controls. Each variant was grown in 4 replicates. The nodule counting and dry biomass analysis were performed after harvest.

### 4.4. 16s rRNA and recA, atpD, nodC Genes Amplification, Sequencing and Phylogenetic Analysis

The extraction and purification of the total genomic DNA of rhizobia isolates were carried out according to protocol by using the Quick-DNA Fecal/Soil Microbe Kit. Fresh rhizobia biomass from the Petri plate was used for genomic DNA extraction. Gene amplification reactions were performed in a 20 μL volume containing DreamTaq Green PCR Master Mix (2X) 10 μL, 0.1 mg/mL of bovine serum albumin 2.0 μL, 0.2 μL of each 10 μM-primer (Table 2), 5.6 μL of DNA-free water and 2 μL of the isolated DNA.

The 16S rRNA PCR amplification was carried out in a PCR thermal cycler (Bio-Rad My cycler, Berkeley, CA, USA) using a hot-start procedure at 94 °C for 5 min. Conditions consisted of 35 denaturation cycles at 94 °C for 30 s, annealing at 55 °C for 30 s and extension at 72 °C for 90 s, followed by a final extension step of 72 °C for 7 min. *RecA*, *atpD* and *nodC* PCR amplification was performed as described above with the exceptions of primer concentrations (0.8 μL of each primer) and a smaller amount of DNA-free water (4.4 μL). Conditions consisted of 35 denaturation cycles at 95 °C for 30 s, annealing at 55 °C for 30 s and extension at 72 °C for 1 min 30 s, followed by a final extension step of 72 °C for 7 min.

A GeneJet PCR Purification Kit was used for PCR amplicon purification. Pure PCR products were subsequently sequenced by Applied Biosystems 3730XL DNA Analyzer using the same primer set used in PCR amplification.

All sequences were processed and aligned by using BioEdit v.7.2 [74]. Phylogenetic sequence analyses of 16S rRNA, *atpD*, *recA* and *nodC* genes were performed using the MEGA 11.0 software package [75,76]. Phylogenetic analyses were performed using the maximum-likelihood algorithm and selecting the best model for each phylogenetic analysis. All positions containing gaps and missing data were eliminated (complete deletion option). *atpD* and *recA* gene sequences were concatenated and aligned. Kimura 2 [77] and Tamura 3 parameter models [78] were used for 16S rRNA, and the *nodC* phylogenetic analysis and Tamura-Nei [79] parameter model were used for *atpD* and *recA* concatenated and aligned phylogenetic sequence analysis.

### 4.5. Statistical Analysis

The statistical analyses were performed using the statistical software SAS 9.4 (SAS Institute, Cary, NC, USA) [80]. Correlation and one-way analysis of variance (ANOVA) statistical tests were used for data analysis. Tukey’s HSD test was applied to analyze mean comparisons between different variants. The smallest significant difference was calculated using a probability level of *p* ≥ 0.05. The values marked with the same letter showed no significant difference at *p* ≥ 0.05.

## Figures and Tables

**Table 1:** Soil properties of the field where different pea genotypes were grown.

	Properties					
Sample	Granulometric Composition	Humus, %	pH	Nmin, mg kg^−1^	Mobile Phosphorus P_2_O_5_, mg kg^−1^	Mobile Potassium K_2_O, mg kg^−1^
SOIL15, 0–30 cm	Heavy loam	2.84	6.8	9.82	250	240

**Table 2:** Primers used for molecular analysis of rhizobia isolates.

Gene	Primers		Reference
	Forward	Reverse	
16 rRNA	27F 5′-(AGAGTTGATCMTGGCTCAG)-3	1387R 5′-(GGGCGGWGTGTACAAG GC)-3′	[71]
*RecA*	5′-(CGKCTSGTAGAGGAYAAATCGGTGGA)-3′	RecA555r 5′-(CGRATCTGGTTGATGAAGATCACCAT)-3′	[72]
*AtpD*	AtpD273f 5′-(SCTGGGSCGYATCMTGAACGY)-3′	AtpD771r 5′-(GCCGACACTTCCGAACCNGCCTG)-3′	[72]
*NodC*	NodCF 5′-(AYGTHGTYGAYGACGGTTC)-3′	NodCI 5′-(CGYGACAGCCANTCKCTATTG)-3′	[73]

## Data Availability

Data are contained within the article.
